# Endometriosis in infertile women: an observational and comparative study of quality of life, anxiety, and depression

**DOI:** 10.1186/s12905-024-03080-5

**Published:** 2024-04-23

**Authors:** Lilian Pagano Mori, Victor Zaia, Erik Montagna, Fabia Lima Vilarino, Caio Parente Barbosa

**Affiliations:** 1 Programa de Pós-graduação em Ciências da Saúde, Centro Universitário FMABC, Av. Lauro Gomes, Santo André, 2000, 09060-870 SP Brazil; 2 Instituto Ideia Fertil de Saúde Reprodutiva, , Santo Andre - SP, Brasil

**Keywords:** Anxiety, Depression, Endometriosis, Infertility, Quality of life

## Abstract

**Background:**

A women’s chances of getting pregnant decreases in cases of infertility, which may have several clinical etiologies. The prevalence of infertility is estimated as 10–15% worldwide. One of the causes of infertility is endometriosis, defined as the presence of an endometrial gland and/or stroma outside the uterus, inducing a chronic inflammatory reaction. Thus, infertility and endometriosis are diagnoses that significantly affect women’s mental health. This study accessed and compared the levels of depression, anxiety, and quality of life in infertile women with and without endometriosis.

**Methods:**

was an observational and cross-sectional study which included 201 infertile women, 81 of whom were also diagnosed with endometriosis. The STROBE Guidelines was used. The data were collected using validated scales: Hamilton D Questionnaire, Beck Depression Inventory, and Fertility Quality of Life Questionnaire; The data were collected at the Ideia Fertil Institute (Santo Andre, Brazil), between February 28 and June 8, 2019.

**Results:**

the infertile women with endometriosis reported higher presence of depressive symptoms and a lower quality of life compared to women with infertility only. Similar presence of anxiety symptoms was observed regardless of being diagnosed with endometriosis. Women with infertility and endometriosis presented lower levels in quality-of-life domains when compared to women with infertility only - Mind and Body (58.33 × 79.17, *p* < 0.001), Relational (75 × 81.25, *p* = 0.009), Social (66.67 × 77.08, *p* = 0.001), Emotional (50.62 × 67.43, *p* < 0.001).

**Conclusion:**

the findings indicate the need for increased psychosocial support care for women suffering from infertility and endometriosis to assist them in maintaining and managing their own mental health and achieving their reproductive goals.

## Background

Estimates show that healthy young women, under 25 years old, have the best chances of becoming pregnant, with a progressive decline in fertility ranging from 4.5% (25 years old) to 100% (50 years old) [1]. However, this percentage decreases greatly in cases of infertility, which may have several clinical etiologies [2]. The prevalence of infertility is estimated as 10–15% worldwide [3]. One of the causes of infertility is endometriosis, defined as the presence of an endometrial gland and/or stroma outside the uterus, inducing a chronic inflammatory reaction [4], with a prevalence ranging from 5 to 10% among women of reproductive age [5]. .

Women with infertility lose control over reproductive decisions and experience feelings of guilt, sadness, shame, and social isolation [6, 7]. These feelings reduce quality of life and negatively affect mental health [8, 9]. The relationship between endometriosis and infertility is expressive, about 40% of women with endometriosis are infertile, and between 25% and 50% of infertile women have endometriosis [10]. In addition, clinical symptoms of endometriosis such as menstrual irregularity, chronic pelvic pain (CPP), dysmenorrhea, and dyspareunia can emotionally affect patients [11, 12].

Some of the disorders associated with endometriosis include depression and anxiety [12, 13]. A meta-analysis indicated that the magnitude of the difference in the occurrence of these two symptoms between healthy women and those with endometriosis is 0.71 for depression and 0.60 for anxiety, with both showing greater prevalence in the group of women with endometriosis [14]. Another study conducted in the United Kingdom with data from 202,276 women found that the group with endometriosis had a higher prevalence of depression (9.8%) and anxiety (3.6%) compared to the group of healthy women [15]. Additionally, endometriosis can impair women’s functional capacity [16], particularly in cases with dyspareunia [17].

Consequently, women diagnosed with endometriosis experience a reduction in quality of life (QoL) [18–20], which is defined as an individual’s perception of their own life, taking into account their cultural background, values, aspirations, and expectations [21–23]. A study [24] comparing QoL levels between healthy women and those with endometriosis revealed an average decrease of 30 points in QoL among participants with endometriosis.

Previous studies [9, 11–13, 25] demonstrated that endometriosis and infertility negatively affect QoL and favor increased levels of anxiety and depression. To enable a more personalized and specific understanding of this demographic, this study uniquely identified and compared anxiety, depression, and QoL levels among infertile women both with and without endometriosis, while also examining the correlations between these variables.

## Methods

### Participants and setting

This was an observational and cross-sectional. Internationally validated and self-applicable scales were used. This study used the STROBE [26] for the reporting of observational studies.

Sample size was calculated using the G*Power software, a significance value of 5% and a minimum test power of 95% were used. The analysis indicated a minimum of 71 participants per group. A larger number of participants were invited to ensure the minimum number was met, accounting for possible participant loss. The participants were subdivided into two groups: Comparator group (A): 120 patients with infertility diagnosis only, and Endometriosis group (B): 81 patients with infertility and endometriosis diagnosed by video-laparoscopy and confirmed with histopathology. Patients included were at the earlier stage of the treatment, after the first consultation or during the clinical testing before the first ovulatory induction cycle and in their first assisted reproduction treatment.

This study was conducted at the Ideia Fertil Reproductive Health Institute, located in São Paulo, Brazil. The sample was characterized as non-probabilistic type. The data were collected between February 28 and June 8, 2019. A total of 230 women were invited to participate. However, 29 of these women declined their participation, indicating no interest or no time. There were 201 infertile women who met the inclusion criteria: [1]age equal to or above 18 years and [2]diagnosis of infertility. The exclusion criteria were: [1]diagnosis of a psychiatric disorder [2], psychotherapy in the last six months [3], psychotropic medication in the last six months [4], history of fibromyalgia [5], neuropathy [6], osteopathy, and [7]presence of malignant tumors. The participants were invited in person and individually exclusively by the author LPM to reduce possible biases while they waited for a medical consultation at that Institute.

### Measures

Sociodemographic Questionnaire - developed ad-hoc for this study, included questions to characterize the participants, such as age, partner’s age, infertility time.

Fertility Quality of Life (FertiQol) [23] − 26 items in four domains: Mind-Body, Relational, Social, and Emotional. The answers are on a five-point Likert scale. Higher scores mean higher QoL. The Brazilian version utilized in this study is official and accessible on the authors’ website (Cardiff University), which was adapted from the Portuguese language validation process [27]. Cronbach’s Alpha of the Fertiqol was 0.921.

Hospital Anxiety and Depression Scale (HADS), validated in Brazilian Portuguese [28] − 14 items, seven of which cover anxiety symptoms and seven cover depression. Each question is scored on a scale (0–3), composing a maximum score of 21 points for each scale. Higher scores indicate higher levels of anxiety and depression, and the scale has a cutoff: up to or equal to seven points indicates no anxiety/depression, and eight or higher points indicates the presence of anxiety/depression. Cronbach’s Alpha of the HADS (alpha = 0.809).

Beck Depression Inventory II (BDI-II), validated in Brazilian Portuguese [29] - measures depressive symptoms and consists of 21 items, each corresponding to a specific category of symptoms and attitude, such as sadness, pessimism, loss of pleasure, guilty feelings, and other aspects [23]. Each question is scored on a scale (0–3), with a total score ranging from 0 to 63. A score of 0–10 points indicates no depressive symptoms, 11–63 points indicates the presence of depressive symptoms. Cronbach’s Alpha of the BDI (alpha = 0.877).

### Statistical analysis

R 4.2.1 used for data transcription and analysis. Basic and Psych Packages were performed. The data were independently typed by two researchers (LPM and VZ) and then combined to avoid transcription errors. Missing data were checked and not found. The distribution of normality of continuous variables was verified using the Kolmogorov-Smirnov test. For the aim of identifying and describing the sample, levels of anxiety, depression, and QoL, we conducted descriptive statistical analyses (e.g., percentile, mean/median) for each group (A and B).

Reliability measures of the psychometric scales were verified using the Cronbach’s Alpha with a rigorous value (cutoff ≥ 0.80) [30], which indicated the exclusion of the depression dimension in HADS (alpha = 0.787).

For comparing variables between groups, we conducted the chi-square test for categorical variables (e.g., presence of anxiety and group) and the Mann-Whitney U test for subgroups comparison (A and B). The FertiQoL emotional domain was the only variable showing a normal distribution, for which the t-test was applied to compare groups. Additionally, to explore correlations between study variables, we conducted Spearman correlation analysis (for continuous scoring of psychometric variables – QoL, anxiety, and depression).

A significance value of 5% was used. Correlation and Cohen coefficient values were considered as small (< 0.30), medium (0.30–0.49), or large (≥ 0.50) [31].

## Results

The population was subdivided into two groups: 120 patients (59.7%) were allocated to group A (with exclusive diagnosis of infertility) and 81 patients (40.3%) to group B (with diagnosis of infertility and endometriosis). The groups were homogeneous for all sociodemographic variables tested: age (34.61 ±4.78), infertility time (4.43 ±3.11), partner’s age (36.71 ±6.31) and primary infertility (90.5%) (Table 1).

**Table 1:** Clinical and demographic characteristics of the participants (comparative)

Variable	Group A (*n* = 120)	Group B (*n* = 81)	Mann-Whitney (p)
	Mean(SD)	Mean(SD)	
Age (years)	34.35(5.39)	34.99(3.69)	0.591
Infertility (years)	4.54(3.53)	4.27(2.37)	0.477
Age partner (years)	36.95(7.14)	35.35(4.83)	0.938
	**n(%)**	**n(%)**	**Chi-square (p)**
Primary Infertility	106 (88.3)	76 (93.8)	0.192

SD = Standard Deviation; Group A: patients with infertility diagnosis only; Group B: patients with Endometriosis and infertility

A significant difference was observed between groups A and B for levels of depressive symptoms (*p* = 0.002) and anxiety (*p* = 0.026), being greater for group B, (infertility and endometriosis). Both groups showed statistically significant differences in relation to QoL, with group A having better levels in all areas of QoL. Moreover, the effect sizes between the groups were significant, except for anxiety, indicating a medium effect for depression (higher levels in Group B), QoL Relation and Social (both with higher scores for Group A), and a large effect for QoL Mind and Body, and Emotional (both with higher scores in Group A) – as shown in Table 2.

**Table 2:** Comparison between groups and depression, anxiety, and quality of life

Variables	Group A (*n* = 120)	Group B (*n* = 81)	*p*	Cohen d	*p*
	n (%)	n (%)	Chi-square		
**Anxiety**				-0.2292	
Absent	85 (70.8)	45 (55.6)	0.026		0.055
Present	35 (29.2)	36 (44.4)			
**Depression**				-0.463	< 0.001
Absent	100 (75.6)	52 (64.2)	0.002		
Present	20 (16.7)	29 (35.8)			
	**Median (Interquartile range)**		**Mann-Whitney U**		
**Domains Quality of Life**					
Mind and Body	79.17 (25.00)	58.33 (42.00)	< 0.001	0.8482	< 0.001
Relation	81.25 (24.00)	75.00 (23.00)	0.009	0.3465	0.009
Social	77.08 (29.00)	66.67 (31.00)	0.001	0.4623	0.001
	**Mean (Standard Deviation)**		**T-Test**		
Emotional	67.43 (22.49)	50.62 (22.77)	< 0.001	0.7437	< 0.001

Group A: patients with infertility diagnosis only; Group B: patients with Endometriosis and infertility

Correlations between the psychometric variables studied were verified, all of which were significant (*p* ≤ 0.001), indicating inverse correlations of moderate level between the relational domain in FertiQoL and anxiety (rho= -0.360) and depression (rho= -0.412), and between the social domain in FertiQoL and anxiety (rho= -0.420). The other correlations between depression, anxiety, and the domains of QoL remained inverse and strong. Depression and anxiety were positively highly correlated (rho = 0.620).

Considering the division between Group A and B, a stronger inverse correlation between depressive symptoms and quality of life is observed in the group with endometriosis compared to the group with infertility only (Table 3).

**Table 3:** Spearman correlation between BDI-II, HAD and quality of life domains

		Geral		Group A		Group B	
		BDI-II	HAD Anxiety	BDI-II	HAD Anxiety	BDI-II	HAD Anxiety
**HAD Anxiety**		0.620^***^	-	0.605^***^	-	0.584^***^	-
**Domain FertiQoL**	Emotional	− 0.628^***^	− 0.510^***^	− 0.543^***^	− 0.467^***^	− 0.637^***^	− 0.497^***^
	Mind and Body	− 0.599^***^	− 0.501^***^	− 0.515^***^	− 0.494^***^	− 0.589^***^	− 0.423^***^
	Relational	− 0.412^***^	− 0.360^***^	− 0.352^***^	− 0.345^***^	− 0.450^***^	− 0.359^***^
	Social	− 0.558^***^	− 0.420^***^	− 0.483^***^	− 0.427^***^	− 0.560^***^	− 0.357^***^

## Discussion

### Summary of findings

This study measured QoL, and depressive and anxiety symptoms in women with infertility, verifying the possible impact between the psychological variables and the double diagnosis: infertility and endometriosis. The findings indicate that women with an overlapping diagnosis (endometriosis-infertility) have higher levels of depressive symptoms and lower QoL than women with infertility only. In addition, lower QoL levels were related to higher levels of anxiety and depressive symptoms.

Data from the literature suggests that sociodemographic variables (e.g., age, infertility duration, partner’s age, and type of infertility) may influence QoL, anxiety, and depression [24, 32, 33], potentially introducing confounding factors in the psychometric measures used [34, 35]. However, since our groups did not show differences in these variables, we suggest that they may not have been determining factors for the differences found in this study.

The levels of depressive symptoms found were higher than those in the general population, estimated at 4.4% according to a study by the World Health Organization [16], corresponding to less than a quarter of the presence of depressive symptoms in the population studied. Furthermore, higher levels of depressive symptoms were observed in participants with both diagnoses: endometriosis and infertility. A similar result was found in a study involving women with endometriosis, which demonstrated a correlation between depression and various comorbidities, including infertility, indicating a stronger link between depressive symptoms and the diagnosis of infertility in women with endometriosis than with other morbidities [36]. Additionally, such findings may be corroborated by the influence of clinical symptoms of endometriosis, beyond infertility, on an individual’s mental health [19, 20, 23].

QoL levels in both groups were lower than those of the general population, consistent with previous studies examining the impact of infertility on QoL [37, 38]. Specifically, lower QoL levels were observed in infertile participants with endometriosis compared to infertile women without endometriosis, aligning with prior research that identifies endometriosis as a factor exacerbating the decline in quality of life and mental health [3, 8, 32, 39–42]. This further supports a trend in the group with infertility alone towards higher quality of life and reduced levels of depressive and anxiety symptoms, as evidenced in this study through Cohen’s d, when contrasted with the group of women with endometriosis and infertility.

Endometriosis and infertility are associated with clinical conditions that cause emotional morbidity, affecting social, sexual, and professional lives [4, 43]. The unregulated immune and inflammatory reactions of endometriosis, which generate CPP, may explain a higher QoL decrease, and more depressive symptoms compared to women with only infertility [12]. To partially restore this impairment, clinical or surgical treatment has proven to be effective in relieving pain [32], but emotional aspects must also be respected and treated by specialists, such as psychologists [20].

Another potential explanation for the correlation between low QoL and mental health in the group with endometriosis and infertility is the connection of endometriosis with psychological factors [33, 36], such as perceived pain and stress, sleep quality [33, 44], anxiety, and depression [37, 45].

Moderate correlations were found between the emotional domain of QoL and depression, with a stronger correlation observed in the infertility with endometriosis group compared to the infertility group alone. This reinforces the connection between impaired mental health and reduced quality of life in situations of heightened anxiety and depression, as seen in infertile women with endometriosis. The findings also indicate that higher levels of anxiety and depression are linked to lower QoL, consistent with previous studies investigating these variables [12, 25, 45].

There are few studies [19, 25, 36] that address psychological aspects in women with both infertility and endometriosis diagnoses, and this study contributes to that field. The data indicates that infertile women with endometriosis exhibit more severe depressive symptoms, anxiety, and decreased quality of life compared to women solely diagnosed with infertility.

### Clinical implications

These results emphasize the relevance of patient-centered education and psychological support for women struggling with endometriosis and infertility to help them manage possible mental health problems and achieve their reproductive goals successfully [13, 45]. Thus, is it possible to question what changes in the reproductive treatment routine which may provide support to patients with infertility and endometriosis. Based on our findings, one of the possibilities would be to include a psychologist in the reproductive team to support patients in maintaining or re-establishing their mental health.

### Strengths and limitations

Some limitations of the present study need to be discussed. First, the study population comprised women diagnosed with infertility and endometriosis at various time intervals since diagnosis. This variation could potentially influence the overall levels of anxiety, quality of life, and depression examined [8, 37], thereby limiting the generalizability of findings to similar populations. Future studies should differentiate the time of diagnosis of each participant. Second, the numerical difference between the groups of infertile women with and without endometriosis is a limiting factor, which hinders comparisons between the two sub-samples. However, the statistical tests used account for these differences in sample sizes, as well as satisfying the minimum sample size outlined by the power calculation.

The use of validated instruments to measure QoL, anxiety and depression in patients is an important strength of the present study, allowing a robust and internationally comparable measurement [11, 18, 46, 47], which is important particularly when considering populations with higher vulnerability to psychiatric disorders such as individuals with infertility [29, 30, 37, 48] and endometriosis [23, 28, 39]. Another important aspect of this study was differentiating the variables studied for the diagnostic overlap between endometriosis and infertility, which improves the knowledge of the emotional aspects of these populations, considering the high co-occurrence of infertility and endometriosis [10]. Additionally, the findings provide information that supports better emotional support and care in reproductive treatment.

## Conclusion

In conclusion, QoL in infertile women is impaired by increased depressive symptoms and anxiety. Compared to women exclusively diagnosed with infertility, infertile women with endometriosis are characterized by a significantly worse emotional state in terms of depressive symptoms and QoL. This suggests the need for care and emotional support in infertility management, especially when associated with endometriosis.

## Data Availability

The data of the present study can be requested from the correspondence author.

## Declarations

### Authors’ information

LPM is an obstetric gynecologist, specialized in Assisted Human Reproduction (AHR) and has a master’s in science in Health Science. VZ is a psychologist, a specialist in psychometrics, data analysis and has a PhD in Health Psychology. EM is a pharmacist and biochemist, specialist in health data analysis, holds a PhD in Biological Science with an emphasis on education. FLV is an obstetric gynecologist, specialized in AHR and holds a PhD in Health Science. CPB is an obstetric gynecologist, specialized in AHR and holds a PhD in Medicine.

## Abbreviations

BDI: II-Beck Depression Inventory II
CPP: chronic pelvic pain
FertiQol: Fertility Quality of Life
HADS: Hospital Anxiety and Depression Scale
QoL: Quality of Life

## References

[1] Vander Borght M, Wyns C (2018). Fertility and infertility: definition and epidemiology. Clin Biochem.

[2] Tarin, Garcia-Perez, Hamatani, Cano (2015). Infertility etiologies are genetically and clinically linked with other diseases in single meta-diseases. Reproductive Biology Endocrinology: RB&E.

[3] WHO WHO. Reproductive health indicators: guidelines for their generation, interpretation and analysis for global monitoring. 2006.

[4] Greil AL, Slauson-Blevins KS, Lowry MH, McQuillan J. Concerns about treatment for infertility in a probability-based sample of US women. J Reprod Infant Psychol. 2019:1–9. 10.1080/02646838.2019.1587395 PMC6754327 30892066

[5] Taylor HS, Kotlyar AM, Flores VA (2021). Endometriosis is a chronic systemic disease: clinical challenges and novel innovations. Lancet (London England).

[6] Kamel RM (2010). Management of the infertile couple: an evidence-based protocol.

[7] Rockliff HE, Lightman SL, Rhidian E, Buchanan H, Gordon U, Vedhara K (2014). A systematic review of psychosocial factors associated with emotional adjustment in in vitro fertilization patients. Hum Reprod Update.

[8] Chachamovich J, Chachamovich E, Fleck MP, Cordova FP, Knauth D, Passos E (2009). Congruence of quality of life among infertile men and women: findings from a couple-based study. Hum Reprod (Oxford England).

[9] G PG, MG VH CVMZ. C C, CP B. Sexual Satisfaction among Involuntarily Childless Women: A Cross-Cultural Study in Italy and Brazil. 2016. 10.1080/03630242.2016.1267690 27922291

[10] Evans MB, Decherney AH (2017). Fertility and endometriosis. Clin Obstet Gynecol.

[11] Young K, Fisher J, Kirkman M (2017). Clinicians’ perceptions of women’s experiences of endometriosis and of psychosocial care for endometriosis. Aust N Z J Obstet Gynaecol.

[12] Lagana AS, La Rosa VL, Rapisarda AMC, Valenti G, Sapia F, Chiofalo B (2017). Anxiety and depression in patients with endometriosis: impact and management challenges. Int J Womens Health.

[13] Facchin F, Barbara G, Dridi D, Alberico D, Buggio L, Somigliana E (2017). Mental health in women with endometriosis: searching for predictors of psychological distress. Hum Reprod (Oxford England).

[14] van Barneveld E, Manders J, van Osch FHM, van Poll M, Visser L, van Hanegem N (2022). Depression, anxiety, and correlating factors in endometriosis: a systematic review and Meta-analysis. J Womens Health (Larchmt).

[15] Koller D, Pathak GA, Wendt FR, Tylee DS, Levey DF, Overstreet C (2023). Epidemiologic and genetic associations of Endometriosis with Depression, anxiety, and eating disorders. JAMA Netw Open.

[16] WHO WHO (2017). Depression and other Common Mental disorders: Global Health estimates.

[17] Pessoa de Farias Rodrigues M, Lima Vilarino F, de Souza Barbeiro Munhoz A, da Silva Paiva L, de Alcantara Sousa LV, Zaia V (2020). Clinical aspects and the quality of life among women with endometriosis and infertility: a cross-sectional study. BMC Womens Health.

[18] Aarts JW, van Empel IW, Boivin J, Nelen WL, Kremer JA, Verhaak CM (2011). Relationship between quality of life and distress in infertility: a validation study of the Dutch FertiQoL. Hum Reprod (Oxford England).

[19] Mori L, Vilarino F, Pádua M, Zaia V, Barbosa C. Depression and quality of life in patients with endometriosisand infertility. Fertil Steril. 2017.

[20] Brasil DL, Montagna E, Trevisan CM, La Rosa VL, Lagana AS, Barbosa CP (2020). Psychological stress levels in women with endometriosis: systematic review and meta-analysis of observational studies. Minerva Med.

[21] WHOQOL-GROUP. The World Health Organization Quality of Life assessment (WHOQOL): position paper from the World Health Organization. Social science & medicine. (1982). 1995;41(10):1403-9. 10.1016/0277-9536(95)00112-k 8560308

[22] Fleck MP, Louzada S, Xavier M, Chachamovich E, Vieira G, Santos L (2000). [Application of the Portuguese version of the abbreviated instrument of quality life WHOQOL-bref]. Rev Saude Publica.

[23] Boivin J, Takefman J, Braverman A (2011). The fertility quality of life (FertiQoL) tool: development and general psychometric properties. Hum Reprod (Oxford England).

[24] Rossi V, Tripodi F, Simonelli C, Galizia R, Nimbi FM (2021). Endometriosis-associated pain: a review of quality of life, sexual health and couple relationship. Minerva Obstet Gynecol.

[25] Wu MH, Su PF, Chu WY, Lin CW, Huey NG, Lin CY (2021). Quality of life among infertile women with endometriosis undergoing IVF treatment and their pregnancy outcomes. J Psychosom Obstet Gynaecol.

[26] von Elm E, Altman DG, Egger M, Pocock SJ, Gøtzsche PC, Vandenbroucke JP (2014). The strengthening the reporting of Observational studies in Epidemiology (STROBE) Statement: guidelines for reporting observational studies. Int J Surg.

[27] Melo C, Gameiro S, Canavarro MC, Boivin J. Does the FertiQoL assess quality of life? Results from the validation of the Portuguese version of the FertiQoL. Hum Reprod. 2012;27.

[28] Freire MÁ, Figueiredo VLMd, Gomide A, Jansen K, Silva RA (2014). Da MPdS. [Hamilton Scale: study of the psychometric characteristics in a sample from Southern Brazil]. Jornal Brasileiro De Psiquiatria.

[29] Gomes-Oliveira MH, Gorenstein C, Lotufo Neto F, Andrade LH, Wang YP (2012). Validation of the Brazilian Portuguese version of the Beck Depression Inventory-II in a community sample. Braz J Psychiatry.

[30] Streiner D, Norman G, Cairney J. Health measurement scales: a practical guide to their development and use (5th edition). Australian and New Zealand journal of public health. 2016;40(3):294-5. 10.1111/1753-6405.12484 27242256

[31] Cohen J (2013). Statistical power analysis for the behavioral sciences.

[32] Matasariu RD, Mihaila A, Iacob M, Dumitrascu I, Onofriescu M, Crumpei Tanasa I (2017). Psycho-Social aspects of Quality of Life in women with endometriosis. Acta Endocrinol (Buchar).

[33] Rees M, Kiemle G, Slade P. Psychological variables and quality of life in women with endometriosis. J Psychosom Obstet Gynaecol. 2020:1–8. 10.1080/0167482X.2020.1784874 32706632

[34] Liu J, Brodley CE, Healy BC, Chitnis T (2015). Removing confounding factors via constraint-based clustering: an application to finding homogeneous groups of multiple sclerosis patients. Artif Intell Med.

[35] Choi BY (2021). Instrumental variable estimation of truncated local average treatment effects. PLoS ONE.

[36] Skegro B, Bjedov S, Mikus M, Mustac F, Lesin J, Matijevic V (2021). Endometriosis, Pain and Mental Health. Psychiatria Danubina.

[37] Terzioglu F, Turk R, Yucel C, Dilbaz S, Cinar O, Karahalil B (2016). The effect of anxiety and depression scores of couples who underwent assisted reproductive techniques on the pregnancy outcomes. Afr Health Sci.

[38] Ashraf DM, Ali D, Azadeh DM (2014). Effect of infertility on the quality of life, a cross- sectional study. J Clin Diagn Research: JCDR.

[39] Chen LC, Hsu JW, Huang KL, Bai YM, Su TP, Li CT (2016). Risk of developing major depression and anxiety disorders among women with endometriosis: a longitudinal follow-up study. J Affect Disord.

[40] Pope CJ, Sharma V, Sharma S, Mazmanian D (2015). A Systematic Review of the Association between Psychiatric Disturbances and endometriosis. Journal of obstetrics and gynaecology Canada: JOGC = Journal d’obstetrique. et gynecologie du Can : JOGC.

[41] Kocas HD, Rubin LR, Lobel M (2023). Stigma and mental health in endometriosis. Eur J Obstet Gynecol Reprod Biol X.

[42] Thiel PS, Bougie O, Pudwell J, Shellenberger J, Velez MP, Murji A. Endometriosis and mental health: a population-based cohort study. Am J Obstet Gynecol. 2024. 10.1016/j.ajog.2024.01.023 38307469

[43] Cavaggioni G, Lia C, Resta S, Antonielli T, Benedetti Panici P, Megiorni F (2014). Are mood and anxiety disorders and alexithymia associated with endometriosis? A preliminary study. Biomed Res Int.

[44] Jia SZ, Leng JH, Shi JH, Sun PR, Lang JH. Health-related quality of life in women with endometriosis: a systematic review. J Ovarian Res. 2012;5(1). 10.1186/1757-2215-5-29 PMC3507705 23078813

[45] Massarotti C, Gentile G, Ferreccio C, Scaruffi P, Remorgida V, Anserini P (2019). Impact of infertility and infertility treatments on quality of life and levels of anxiety and depression in women undergoing in vitro fertilization. Gynecol Endocrinology: Official J Int Soc Gynecol Endocrinol.

[46] Zigmond AS, Snaith RP (1983). The hospital anxiety and depression scale. Acta Psychiatrica Scandinavica.

[47] Thomas FS, Stanford JB, Sanders JN, Gurtcheff SE, Gibson M, Porucznik CA (2015). Development and initial validation of a fertility experiences questionnaire. Reprod Health.

[48] Raguz N, McDonald SW, Metcalfe A, O’Quinn C, Tough SC (2014). Mental health outcomes of mothers who conceived using fertility treatment. Reprod Health.

